# Assessing the Efficacy of Planmeca ProMax® 3D Cone-Beam CT Machine in the Detection of Root Fractures With Varied Metal Artifact Reduction Settings and Three Kilovoltage Peak Levels

**DOI:** 10.7759/cureus.35647

**Published:** 2023-03-01

**Authors:** Ahmed Almeshari, Ahmed Z Abdelkarim, Hassem Geha, Asma A Khan, Nikita Ruparel

**Affiliations:** 1 Department of Oral and Maxillofacial Surgery and Diagnostic Sciences, Najran University, Najran, SAU; 2 Division of Oral and Maxillofacial Radiology, The Ohio State University College of Dentistry, Columbus, USA; 3 Department of Comprehensive Dentistry, Oral and Maxillofacial Radiology Program, University of Texas Health Science Center at San Antonio School of Dentistry, San Antonio, USA; 4 Department of Endodontics, University of Texas Health Science Center at San Antonio School of Dentistry, San Antonio, USA

**Keywords:** accuracy, root fracture, kvp, cbct, metal artifact

## Abstract

Background: This study aims to examine the accuracy of cone-beam computed tomography (CBCT) machines in detecting root fracture when using different metal artifact reduction (MAR) settings at different kilovoltage peak (kVp) levels.

Methodology: Sixty-six tooth roots were treated endodontically using a standardized technique. Of these, 33 roots were randomly selected to be fractured; the other 33 roots were intact and used as controls. The roots were placed randomly in prepared beef ribs to mimic the alveolar bone. Imaging was performed by Planmeca ProMax® 3D (Planmeca, Helsinki, Finland) using different MAR settings (no, low, mid, and high) at three different levels of kVp: 70, 80, and 90. Sensitivity, specificity, and area under the receiver operating characteristic curve (AUC) were calculated.

Results: There was a significant difference in accuracy when using different MAR settings within the group of 70 kVp. Likewise, within the group of 90 kVp. There was no significant difference between different MAR settings at 80 kVp. Using low MAR/90 kVp had significantly higher accuracy relative to other MAR settings at 90 kVp; it also had the highest values of sensitivity, specificity, and AUC in the study. Using mid and high MAR at 70 kVp or 90 kVp decreased accuracy significantly. Mid MAR/90 kVp was the least effective setting in this study.

Conclusions: Using low MAR at 90 kVp significantly increased the accuracy within the group of 90 kVp. In contrast, mid MAR and high MAR in 70 and 90 kVp, respectively, decreased accuracy significantly.

## Introduction

A definitive diagnosis of root fracture (RF) using cone-beam computed tomography (CBCT) is a challenging task in clinical dentistry, especially in the presence of various root canal-filling materials [1]. High-density materials generate beam hardening and scatter effect artifacts [2,3]. Intracanal materials induce such artifacts, reducing the accuracy of CBCT in detecting RF and making the diagnosis difficult [1,4]. Beam hardening artifact occurs when lower energy rays from the beam spectrum are filtered and removed by high-density objects. At the same time, higher energy rays pass beyond absorbing objects, are reconstructed as errors, and are back-projected into the volume as dark bands [2,3].

A metal artifact reduction (MAR) algorithm was introduced as a method to reduce artifacts [5]. Studies have shown that MAR tools improve image quality by increasing the contrast-to-noise ratio (CNR) in the presence of metallic fillings [6-8]. Another method to reduce artifacts is kilovoltage peak (kVp) adjustment; increasing kVp has demonstrated a reduction of CBCT artifacts [7,9]. Some studies have shown no improvement in the accuracy of CBCT in detecting RF when using MAR [10,11].

Also, a study [12] found no significant difference in the ability of CBCT to detect RF when kVp was adjusted between 70 kVp and 74 kVp. However, CBCT machines and MAR algorithms can be improved by manufacturers and tested again at different settings of exposure parameters. The new generation of machines uses novel image-improving algorithms and smaller voxel sizes as well as four MAR settings (no, low, mid, and high). The impact of different combinations of MAR settings and kVp levels on the accuracy of CBCT in detecting RF is not well studied.

## Materials and methods

In this study, we used a phantom of 66 teeth that was used in previous studies at the School of Dentistry at the University of Texas Health Science Center in San Antonio, Texas, United States [10]. Extracted single root teeth were collected, and the crowns were sectioned. The roots were then treated endodontically using the standardized protocol. Thirty-three of the roots were selected randomly to be fractured at different levels of the root using a nail and a hammer until a displaced fracture occurred; then, the two root fragments were glued using the same material in all roots. The roots were placed randomly in prepared beef ribs to mimic the alveolar bone. They were distributed into eight groups; each group having eight or nine roots. The ribs were covered with three layers of wax to mimic gingival tissues, then immersed in a water container to mimic surrounding soft tissue.

Imaging

The Planmeca ProMax® 3D (Planmeca, Helsinki, Finland) was the imaging tool; scans were acquired at four different settings of MAR (no, low, mid, and high) at three settings of kVp: 70, 80, and 90. In all, there were 12 different settings for each group of roots; in addition, 792 root images were evaluated twice, resulting in 1584 observations. The tube current was constant at the high-resolution setting, 10 mA, and a voxel size of 0.15 mm. The volume size was 8 cm × 5 cm.

Scans distribution

The scans were randomly distributed between five observers to avoid observer fatigue. Oral and Maxillofacial Radiology graduate residents who have been trained in CBCT scans and root fracture detection at the same institution, the University of Texas Health Science Center at San Antonio, were asked to rate the roots on a scale of 1 to 5 as: 1 (definitely negative for a fracture), 2 (probably negative for a fracture), 3 (not sure), 4 (probably positive for a fracture), and 5 (definitely positive for a fracture). Before the rating sessions, the evaluation process was explained to all observers. There were two observers assigned to each root in the different settings. For the intra-observer agreement evaluation, a set of duplicate scans was created to be rated by each observer in two different sessions separated by two weeks. Invivo6 software (Anatomage Inc., California, United States) was used for viewing and interpreting the scans. All observers used the same type of monitor in a reading room with dim light.

Statistical analysis

The kappa statistic with a 95% confidence interval was used to evaluate interobserver and intraobserver agreements. The values were interpreted as follows: poor, 0-0.20; fair, 0.21-0.40; moderate, 0.41-0.60; substantial, 0.61-0.80; almost perfect, 0.81-0.99; and perfect, 1.00. To calculate sensitivity and specificity, observer ratings were compared to the actual status of the root to determine how well each imaging modality assisted the observer with correctly identifying a fracture. The observer rating was considered to agree with the actual status of fracture if it was a 4 or 5, and ratings of 1 or 2 were considered to be in agreement with the actual status of no fracture. Ratings of 3 were ignored. Only 54 of 1584 images (3.5%) were rated with a 3. An observer rating that agreed with the actual status of the fracture was a true positive (tp), and agreement with the actual status of no fracture was a true negative (tn). When the observer rating disagreed with the status of the fracture, it was a false negative (fn), and when the rating implied fracture when there was no fracture, it was a false positive (fp) [13]. For sensitivity and specificity, the confidence intervals were calculated using the binomial test of proportions. The area under the receiver-operator curve (AUC) was prepared to evaluate accuracy. The Wilcoxon-Mann-Whitney test was used to calculate the confidence intervals (CIs) for the AUC values [14]. The R statistical computing environment was used for the statistical analysis; the program code developed by Kate Nambiar was modified slightly and used for these calculations (simple receiver-operator curve (ROC) plots with ggplot2. March 21, 2012).

## Results

Interobserver and intraobserver agreements

The kappa values illustrated in Table 1 indicate a perfect intraobserver agreement for observer A, a substantial agreement for observers B and E, and a moderate intraobserver agreement for observers C and D. For interobserver agreement (Table 2), the kappa values for the settings no MAR/70 kVp and low MAR/70 kVp indicate a substantial agreement between the observers evaluating these settings. There was a fair agreement between the observers in the settings mid MAR/70 kVp, high MAR/70 kVp, and no MAR/80 kVp. A moderate agreement was obtained between the observers in the following settings: low MAR/80 kVp, mid MAR/80 kVp, and high MAR/80 kVp. The same level of agreement was obtained in the settings no MAR and high MAR at 90 kVp. A poor agreement between the observers was obtained when using mid MAR at 90 kVp. There was an almost perfect agreement when using low MAR at 90 kVp.

**Table 1:** Intraobserver agreement between observers Poor: 0–0.20; fair: 0.21–0.40; moderate: 0.41–0.60; substantial: 0.61–0.80; almost perfect: 0.81–0.99; and perfect: 1.00.

Observer	Observed Agreement	Kappa (Lower Confidence Level, Upper Confidence Level)
A	1.00	1.00 (1.00, 1.00)
B	0.90	0.78 (0.64, 0.92)
C	0.87	0.51 (0.25, 0.77)
D	0.81	0.60 (0.44, 0.76)
E	0.81	0.61 (0.44, 0.78)

**Table 2:** Interobserver agreement Poor: 0–0.20; fair: 0.21–0.40; moderate: 0.41–0.60; substantial: 0.61–0.80; almost perfect: 0.81–0.99; and perfect: 1.00. MAR: metal artifacts reduction

Kilovoltage Peak (kVp) and MAR Settings		Observed Agreement	Kappa value	95% Confidence Interval (Upper Confidence Level and Lower Confidence Level)	
70	No	0.864	0.714	0.5435	0.8845
70	Low	0.894	0.776	0.6192	0.9328
70	Mid	0.742	0.294	0.0412	0.5468
70	High	0.742	0.255	0	0.5176
80	No	0.697	0.262	0.0111	0.5129
80	Low	0.818	0.583	0.3733	0.7927
80	Mid	0.803	0.546	0.3343	0.7577
80	High	0.758	0.487	0.2714	0.7026
90	No	0.788	0.542	0.3303	0.7537
90	Low	0.955	0.899	0.7873	1.0107
90	Mid	0.621	0.12	0	0.3493
90	High	0.803	0.541	0.3234	0.7586

Sensitivity 

**Figure 1:**
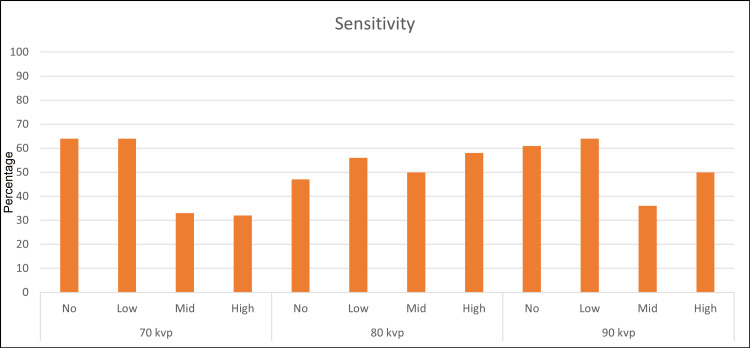
Plot graph for sensitivity.

The sensitivity varies significantly within each group of 70 kVp and 90 kVp when using four different MAR settings (Figure 1) (Table 3). There was no statistically significant difference between the sensitivity values within the group of 80 kVp when using different MAR settings. Within the group of 70 kVp, the sensitivity values of no MAR and low MAR were significantly greater than those of high MAR and mid MAR. Within the group of 90 kVp, using low MAR had greater sensitivity than all the settings within the group with a significant difference from mid and high MAR settings. Mid MAR/90 kVp dramatically decreased sensitivity and obtained the lowest value in the study. Using mid and high MAR at 70 kVp or 90 kVp decreased sensitivity significantly relative to the other settings in each group.

**Table 3:** Sensitivity for three kilovoltage peak (kVp) values (95% confidence interval). Uppercase letters in the table are for values in each row independently; different uppercase letters in a row indicate a significant difference.  Lowercase letters are for all values in the table; Different lowercase letters mean significant difference. MAR: metal artifact reduction

Settings	No MAR	Low MAR	Mid MAR	High MAR
70 kVp	0.64 (0.52, 0.75) A, a	0.64 (0.52, 0.75) A, a	0.33 (0.22, 0.45) B, e	0.32 (0.21, 0.43) B, e
80 kVp	0.47 (0.35, 0.59) A, c, d	0.56 (0.44, 0.68) A, a, b	0.50 (0.38, 0.62) A, b, c	0.58 (0.46, 0.69) A, b, c
90 kVp	0.61 (0.49, 0.72) A, a, b	0.64 (0.52, 0.75) A, a	0.36 (0.25, 0.48) C, d, e	0.50 (0.38, 0.62) B, b, c

Specificity

**Figure 2:**
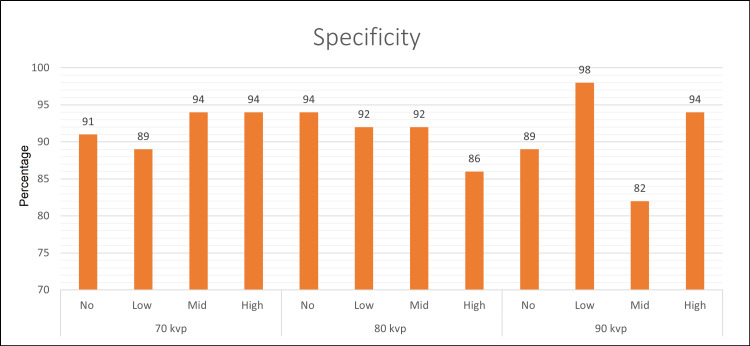
Plot graph for specificity (95% confidence interval)

The specificity values are illustrated in Figure 2 and Table 4. Among all the settings, using the combination of low MAR/90 kVp had a significantly higher specificity value than all settings in the study. At a constant 70 kVp, there was no significant difference between the specificity values when using different MAR settings. However, within the group of 80 kVp, using the no MAR setting had a significantly higher specificity value than high MAR. In the same group, the no MAR, low MAR, and mid MAR settings were not significantly different. In the group of 90 kVp, using low MAR had the highest specificity value among all settings in this study, with a significant difference from all the settings in the study. However, when using mid MAR/90 kVp, specificity was extremely decreased; it was the lowest specificity value among all settings in this study.

**Table 4:** Specificity for three kilovolt peak (kVp) values (95% confidence interval). Uppercase letters in the table are for values in each row independently. Different uppercase letters in a row indicate a significant difference. Lowercase letters are for all values in the table. Different lowercase letters mean a significant difference. * indicates the highest value with a significant difference from all the values in the table. MAR: metal artifact reduction

Settings	No MAR	Low MAR	Mid MAR	High MAR
70 kVp	0.91 (0.84, 0.98) A a, b	0.89 (0.82, 0.97) A, a, b, c	0.94 (0.88, 1.00) A, a	0.94 (0.88, 1.00) A, a
80 kVp	0.94 (0.88, 1.00) A, a	0.92 (0.86, 0.99) A, B a, b	0.92 (0.86, 0.99) A, B, a, b	0.86 (0.78, 0.95) B, b, c
90 kVp	0.89 (0.82, 0.97) B, C, a, b, c	0.98 (0.96, 1.00) A, *	0.82 (0.73, 0.91) C, c	0.94 (0.88, 1.00) B, a

AUC

**Figure 3:**
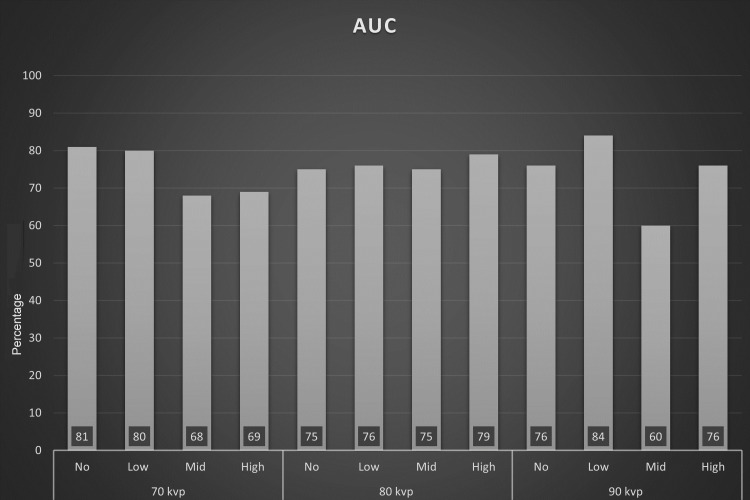
Plot graph for AUC AUC:  area under the receiver operating characteristic curve

The AUC values were obtained to evaluate accuracy; they are explained in Figure 3 and Table 5. There was a significant difference between the four MAR settings within the group of 70 kVp; using no MAR or low MAR had significantly higher accuracy than mid MAR and high MAR. There was no statistical difference between no MAR/70 kVp and low MAR/70 kVp. When using a constant 80 kVp, there was no statistically significant difference between the four MAR settings. However, within the group of 90 kVp, low MAR had the highest AUC value in this study; it was significantly higher than the other MAR settings within the group of 90 kVp. However, using mid MAR/90 kVp obtained the lowest AUC value in the study.

**Table 5:** Area under the receiver operating characteristic curve (95% confidence interval). Uppercase letters are for values is in each row independently; different uppercase letters in a row indicate a significant difference. Lowercase letters are for all values in the table; different lowercase letters mean significant difference.  * indicates the highest value in the table. MAR: metal artifact reduction

	No MAR	Low MAR	Mid MAR	High MAR
70 kVp	0.81 (0.73, 0.88) A, a, b	0.80 (0.72, 0.87) A, a, b	0.68 (0.59, 0.77) B, c, d	0.69 (0.60, 0.78) B, c, d
80 kVp	0.75 (0.66, 0.83) A, b, c	0.76 (0.68, 0.84) A, b, c	0.75 (0.66, 0.83) A, b, c	0.79 (0.71, 0.87) A, a, b
90 kVp	0.76 (0.68, 0.84) B, b, c	0.84 (0.78, 0.91) A, a, *	0.60 (0.51, 0.70) C, d	0.76 (0.68, 0.84) B, b, c

## Discussion

The clinical decision of whether to save or extract a tooth when RF is suspected is often difficult to make. The presence of root canal filling material would make it even more difficult due to the induction of artifacts as a result of x-ray interaction with intracanal high-density fillings [1,12,15,16]. The effectiveness of using MAR to improve the image quality of CBCT has been studied. Studies have shown that using MAR improves CNR and reduces artifacts [6,8,17-19]. Also, studies have found that increasing kVp and using MAR have a positive impact on CNR and artifact [7,8,20]. Thus, the effect of MAR and kVp on the accuracy of CBCT in detecting RF seems promising. Identifying the best combination of the MAR/kVp settings to detect RF may improve the accuracy with a positive impact on the clinical decision.

In this study, there was a weak interobserver agreement in the mid MAR/90 kVp setting and a fair agreement in the mid MAR/70 kVp, high MAR/70 kVp, and no MAR/80kVp settings. Some other settings obtained moderate agreement, as illustrated in Table 2. These low kappa values in this study are very likely due to the settings that were not properly assisting the observers in detecting RF; the observers were guessing in a significant portion of observations in these settings. This pattern also showed that these settings were the least effective combinations in detecting RF. This variation in kappa values of the interobserver agreement had a similar pattern to what was found in the study by Bechara et al. [10]. 

Some reported studies have shown a negative impact of using MAR on RF detection [10,11]. Our study is slightly different; we evaluated the accuracy of CBCT in detecting RF when using different levels of MAR as no, low, mid, and high MAR at different kVp settings. The highest sensitivity value was obtained in the following combinations: no MAR/70 kVp, low MAR/70 kVp, and low MAR/90 kVp. The best combination of sensitivity, specificity, and AUC was attained when using low MAR/90 kVp; the AUC value of this setting was significantly higher than the other values within the group of 90 kVp. Interestingly, the least effective setting in this study was the setting of mid MAR/90 kVp; it had the lowest value of AUC and specificity as well as a relatively low sensitivity value. Likewise, there was a significant decrease in sensitivity and AUC when mid and high MAR were used in combination with 70 kVp. In the same settings, there was a significant improvement in the specificity, but with a sensitivity trade-off leading to low AUC values. The combinations high MAR/70 kVp, mid MAR/70 kVp, no MAR/80 kVp, and high MAR/90 kVp had high specificity values of 0.94 (95%CI: 0.88-1.00); however, this value was still significantly lower than the highest specificity value when using low MAR/90 kVp (0.98; 95%CI 0.96-1.00). Although low MAR/90 kVp had the highest AUC value (0.84; 95%CI: 0.78-0.91), it was not statistically different from the following combinations: no MAR/70 kVp, low MAR/70 kVp, and high MAR/80 kVp (0.81, 0.80, and 0.79, respectively), but statistically different from the other settings in the study. The AUC values within the group of 80 kVp were not significantly different. The reason is not clear; it may be due to the balance between the MAR level and the kVp effect on artifacts induced by intracanal filling. The AUC value of high MAR/80 kVp was significantly higher than mid MAR/70 kVp, high MAR/70 kVp, and mid MAR/90 kVp.

The results of this study were partially different from the reported studies by Bechara et al. [10] and Bezerra et al. [11]. The differences are probably due to the fact that we used four different levels of MAR at different kVp settings in this study in contrast to one MAR level and one kVp level in their studies. In our study, we found that using low MAR at 90 kVp had significantly higher accuracy than the other MAR settings within the group of 90 kVp. This finding was in disagreement with their studies' findings. However, using mid MAR and high MAR at 70 kVp or 90 kVp decreased the accuracy significantly; this finding was in agreement with the reported studies by Bechara et al [10] and Bezerra et al. [11]. An important finding was noted, when adjusting 70 kVp, 80 kVp, and 90 kVp at no MAR, there was no significant difference; this agreed with Pinto et al. [12].

Hence, understanding the mechanism of the MAR algorithm and how it affects the real image data seem crucial. In 2003, Mahnken et al. [5] introduced a MAR approach using the interpolation technique; the data obtained from the hardened beam were considered unnecessary data and eventually removed from the image. According to Bechara et al. [10], the MAR algorithm in Planmeca ProMax has a threshold; any extremely denser data will be treated as unnecessary data and will be removed, but with a decrease in accuracy. It is essential to know to what extent the MAR tool can modify the data without precluding fine details like a fracture line.

There were some limitations in this study. We used a medium volume size that was relatively large. The volume size was limited by phantom size; the smallest volume that can be used to include the whole phantom was 5-8 cm. Bechara et al. found that small-volume scans have higher CNR than large volumes [21]. In addition, Hassan et al. found that the quality of three-dimensional surface models of dental arches on CBCT was better when using a small volume with a smaller voxel size [22]. Voxel size has a significant impact on evaluating fine details like a fracture line. The option of a small volume, high resolution on the Planmeca ProMax 3D has a voxel size as small as 0.075 mm; this may improve accuracy even more. Therefore, using a smaller phantom to fit the small volume option on the Planmeca ProMax 3D is possible and may lead to more accurate settings. It can be done by decreasing the phantom size and then evaluating in the future. It also important to consider the effect of using MAR on spatial resolution; this is crucial for RF detection and needs to be investigated in future studies as well. 

## Conclusions

Low MAR at 90 kVp increased the accuracy of detecting RF. However, mid MAR and high MAR at 70 kVp or 90 kVp decreased accuracy. There was no significant difference between MAR settings within the group of 80 kVp. Therefore, selecting the appropriate combination of MAR/kVp can improve CBCT accuracy in detecting RF.
